# Groundsel Bush (*Baccharis halimifolia*) Extract Promotes Adipocyte Differentiation In Vitro and Increases Adiponectin Expression in Mature Adipocytes

**DOI:** 10.3390/biology7020022

**Published:** 2018-03-25

**Authors:** Anik Boudreau, Scott Fuller, David M. Ribnicky, Allison J. Richard, Jacqueline M. Stephens

**Affiliations:** 1 Pennington Biomedical Research Center, Louisiana State University Baton Rouge, Baton Rouge, LA 70808, USA; anik.boudreau@pbrc.edu (A.B.); scott.fuller@pbrc.edu (S.F.); allison.richard@pbrc.edu (A.J.R.); 2 School of Kinesiology, University of Louisiana Lafayette, Lafayette, LA 70506, USA; 3 Biotech Center, Rutgers University, New Brunswick, NJ 08901, USA; ribnicky@sebs.rutgers.edu

**Keywords:** fat cells, groundsel bush, botanicals, adiponectin

## Abstract

An ethanolic extract of *Baccharis halimifolia* (groundsel bush, GB), which is a native Louisiana plant with documented use in Creole folk medicine, has been shown to inhibit lipopolysaccharide (LPS)-induced inflammation in cultured macrophages. Here, we examine the effects of GB on adipocyte development and function, as these processes are attractive targets for intervention in insulin resistance. Oil Red O neutral lipid staining, reverse transcription-quantitative polymerase chain reaction (RT-qPCR), and immunoblotting were used to measure GB effects on lipid accumulation, gene expression, and protein abundance, respectively. In differentiating 3T3-L1 adipocytes, GB enhanced lipid accumulation and increased expression of several adipogenic genes (GLUT4, aP2, ADPN, CEBPα, FAS, and PPARγ). Protein levels of two of these adipogenic markers (aP2 and adiponectin) were examined and found to be induced by GB treatment. In mature adipocytes, GB reduced the gene expression of resistin, a pro-inflammatory endocrine factor, increased the adiponectin protein levels in a time-dependent manner, and substantially attenuated the TNF-alpha-induced reduction in adiponectin. In macrophages, GB reduced the expression of pro-inflammatory genes that were induced by LPS. GB produces metabolically favorable changes in differentiating adipocytes, mature adipocytes, and macrophages in vitro, suggesting its potential use as a dietary supplement or nutraceutical to support metabolic health and resiliency.

## 1. Introduction

Metabolic syndrome, which is a collection of risk factors, including insulin resistance and obesity, has emerged as a major public health challenge in the 21st century [1,2]. Adipose tissue, traditionally considered to be little more than a storage site for surplus caloric energy in the form of triglycerides, is now known to play a critical role in maintaining metabolic health [3], and disruptions in adipose tissue development and function contribute to metabolic dysfunction in several ways. For example, we now know that in obesity, adipocyte differentiation is often impaired, and that the inability of adipose tissue to expand is associated with poor metabolic outcomes [4]. In addition to their well-established function as safe storage for lipids, adipocytes are now understood to be indispensable endocrine regulators of insulin sensitivity and glucose homeostasis [5,6], in large part through their capacity to synthesize and secrete adiponectin, which promotes insulin sensitivity in peripheral tissues, such as skeletal muscle and liver, thereby mediating whole-body glucose homeostasis [7]. This endocrine action of adipose tissue is disrupted in obese states [8], which, in turn, further exacerbates the features of metabolic syndrome. Obesity and metabolic disease are also known to be associated with the activation of inflammatory pathways within adipose tissue, where adipocytes and immune cells (principally macrophages) interact and promote insulin resistance, contributing to the vicious cycle of metabolic dysfunction and impaired adipose tissue function.

Nutritional supplements derived from plant sources have a long history of use for medicinal purposes throughout the world, and botanical extracts have been used extensively in drug development. Metformin, which is commonly prescribed to treat type 2 diabetes, is a derivative of a compound from French lilac [9]. Given the importance of adipose tissue in maintaining normal metabolic function, interventions that are aimed at enhancing adipocyte development and secretory function, or at attenuating inflammatory processes in adipocytes or immune cells, have the potential to ameliorate metabolic syndrome and its associated pathologies. To this end, our laboratory has screened a variety of plant extracts for their ability to favorably alter adipocyte development and function. Previous screening efforts have identified an extract of *Artemisia scoparia* as a positive modulator of adipocyte differentiation that was subsequently found to have positive effects on adipose tissue function and on insulin sensitivity in vivo [10,11], underscoring the relevance of adipose tissue as a target for intervention with botanical extracts in metabolic syndrome.

For the present study, in vitro screening efforts identified an extract of *Baccharis halimifolia*, which is commonly known as groundsel bush (GB), as a positive modulator of adipocyte differentiation and function. First, we observed that GB promoted lipid accumulation and enhanced adipogenic gene expression in differentiating 3T3-L1 cells, indicating that it could promote adipocyte differentiation. In mature adipocytes, GB increased adiponectin levels and reduced resistin gene expression, as consistent with a metabolically favorable effect on adipocyte endocrine function. Finally, GB was found to mitigate pro-inflammatory and metabolically harmful changes that were induced by tumor necrosis factor-alpha (TNF-α) in adipocytes or by lipopolysaccharide (LPS) in macrophages. Although documentation exists indicating that groundsel bush has been used in Creole folk medicine in Louisiana, to our knowledge, there are currently no published studies in peer-reviewed sources supporting any medicinal use of this plant. The novel in vitro experiments described in the present study provide evidence that an ethanolic extract of groundsel bush promotes adipocyte differentiation, improves the endocrine profile of mature adipocytes, and mitigates inflammation-related changes known to promote metabolic dysfunction. The data reported herein provide a basis for further investigation of groundsel bush as a nutraceutical or dietary supplement with the potential to favorably alter metabolic function via its effects on adipocytes and macrophages.

## 2. Materials and Methods

### 2.1. Source and Preparation of Groundsel Bush Extract

Plant material was collected and vouchered, and ethanolic extracts were prepared, as described previously [12].

### 2.2. Cell Culture and Treatments

For adipogenesis experiments, murine 3T3-L1 preadipocytes were cultured and grown to confluence, as described previously [11]. Two days after reaching confluence, cells were induced to differentiate using high-glucose Dulbecco’s Modified Eagle’s Medium (DMEM) supplemented with 10% fetal bovine serum (FBS) and standard methylxanthine-dexamethasone-insulin (MDI) cocktail (0.5 mM IBMX (3-isobutyl-1-methylxanthine), 1 μM dexamethasone, and 1.7 μM insulin), or 0.5× MDI cocktail (0.25 mM IBMX, 0.5 μM dexamethasone, and 0.85 μM insulin), plus groundsel bush extract or dimethyl sulfoxide (DMSO) vehicle. 48–72 h after induction, medium was replaced with DMEM plus 10% FBS and 0.425 μM insulin for cells induced with standard MDI cocktail, or 0.21 μM insulin for cells induced with 0.5× MDI cocktail, and cells were treated again with the GB extract. 48 h later, cells were fed with DMEM plus 10% FBS and treated with GB. Cells were harvested or fixed for staining on day 6 post-induction. DMEM, insulin, IBMX, and dexamethasone were obtained from Sigma-Aldrich (St. Louis, MO, USA). Bovine sera were obtained from Hyclone (GE Life Sciences, Logan, UT, USA).

For the experiments using mature adipocytes, preadipocytes were grown and induced to differentiate using standard MDI cocktail, as described above. Treatments were initiated 10–13 days post-induction. For the GB time course, cells were fed every other day with DMEM plus 10% bovine calf serum, and treated every day with GB. For the TNF-α experiment, cells were fed with DMEM plus 0.5% bovine calf serum and were treated with GB and TNF-α every day for three days.

RAW 264.7 macrophages (American Type Culture Collection; Manassas, VA, USA; #TIB-71) were maintained as described previously [12]. For experiments, cells were pre-treated with DMSO vehicle or GB extract for 2 h; LPS was then added to a final concentration of 1 μg/mL for six additional hours.

### 2.3. Lipid Accumulation

Six days after the induction of differentiation, cells were fixed and stained with Oil Red O (ORO), as described previously [11]. ORO staining was quantified by solubilizing the ORO from the stained cells in isopropyl alcohol and measuring absorbance at 520 nm. ORO was obtained from Sigma-Aldrich (St. Louis, MO, USA).

### 2.4. Gene Expression

**Figure 1:**
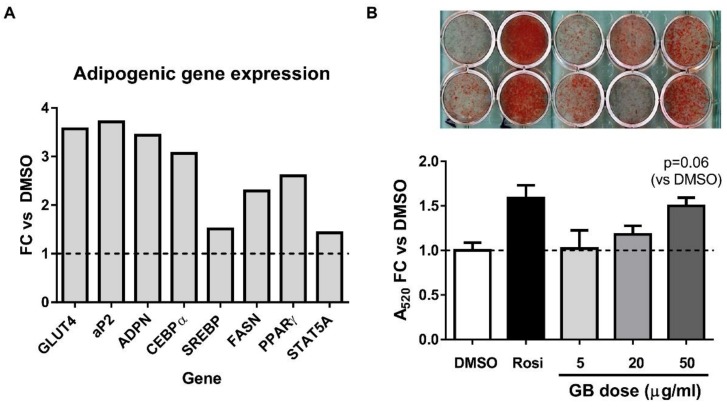
Groundsel bush promotes lipid accumulation and adipocyte marker gene expression in 3T3-L1 cells. (**A**) Six days after 3T3-L1 preadipocytes were induced to differentiate using the standard induction cocktail in the presence or absence of 50 µg/mL of groundsel bush, RNA was isolated, purified, and subjected to RT-qPCR; (**B**) In a separate experiment, 3T3-L1 preadipocytes were induced to differentiate using half-strength induction cocktail containing 5, 20 or 50 µg/mL of groundsel bush (GB). Six days post-induction, cells were fixed and then stained with Oil Red O (photograph in top panel; all of the wells were from the same 12-well plate, and brightness and contrast were equally adjusted for all wells). Staining was quantified by measuring absorbance at 520 nm after extraction of stain in isopropyl alcohol (bottom panel; *n* = 2 wells per treatment). Fold change (FC) was calculated by normalizing the data against the DMSO (dimethyl sulfoxide) vehicle control. Rosiglitazone (Rosi) was used as a positive control in (**B**). Results shown are from duplicate cell culture wells, and the experiment was repeated two additional times on independent batches of 3T3-L1 cells.

Cells were harvested and RNA was purified using the RNeasy Mini kit (Qiagen, Hilden, Germany). RNA was reverse transcribed (RT) using High-Capacity cDNA Reverse Transcription kit (Applied Biosystems, Foster City, CA, USA). For Figure 1, qPCR analysis was conducted using an RT^2^ Profiler custom qPCR array (Qiagen) comprising bench-validated assays for the following genes: adiponectin (ADPN; PPM05260), fatty acid binding protein 4 (aP2) (FABP4; PPM04517), fatty acid synthase (FASN; PPM03816), PPARγ (PPM05108), CCAAT/enhancer binding protein (C/EBP) alpha (CEBPα; PPM04674), Sterol regulatory element binding transcription factor 1 (SREBP1; PPM05094), Solute carrier family 2 (facilitated glucose transporter), member 4 (GLUT4; PPM04166). Peptidyl prolyl isomerase H (PPIH; PPM03699) was included as the reference gene to which all data were normalized. Data analysis was performed using the ΔΔC_T_ method via the PCR Array Data Analysis Web Portal (http://www.sabiosciences.com/pcrarraydataanalysis.php). For all other gene expression assays, qPCR was performed using Takara SYBR premix (Takara Bio USA Inc., Madison, WI, USA) and primers from IDT (Integrated DNA Technologies, Skokie, IL, USA). The reference genes used were: *cyclophilin B* (*ppib*) and *ubiquitin B* (*ubb*). All primer sequences are shown in Table 1.

Assays were performed on the Applied Biosystems 7900HT system using SDS 2.4 software (Applied Biosystems, Foster City, CA, USA), with the following thermal cycling conditions: 2 min, 50 °C; 10 min, 95 °C; 40 cycles of 15 s at 95 °C and 1 min at 60 °C; dissociation stage: 15 s, 95 °C; 15 s, 60 °C; 15 s, 95 °C.

### 2.5. Whole-Cell Extract and Tissue Preparation

Whole-cell extracts were prepared by harvesting adipocyte monolayers in a non-denaturing extraction buffer containing 150 mM NaCl, 10 mM Tris, pH 7.4, 1 mM ethylene glycol tetraacetic acid (EGTA), 1 mM ethylenediaminetetraacetic acid (EDTA), 1% Triton X-100, 0.5% Igepal CA-630, 1 mM phenylmethylsulfonyl fluoride, 1 μM pepstatin, 50 trypsin inhibitory milliunits of aprotinin, 10 μM leupeptin, 1 mM 1, 10-phenanthroline, and 0.2 mM sodium vanadate. Lysates were frozen and thawed, and then passed through a 20-gauge needle three times before being centrifuged at 17,500 g for 10 min at 4 °C. Care was taken to remove the lipid layer, and supernatants were recovered and transferred to fresh tubes. Protein content was determined by BCA protein assay (Sigma-Aldrich, St. Louis, MO, USA).

### 2.6. Gel Electrophoresis and Immunoblotting

Samples were separated on 7.5%, 10% or 15% sodium dodecyl suflate (SDS)-polyacrylamide gels and were transferred to nitrocellulose membranes, which were subjected to standard immunoblotting techniques and infrared detection on the Odyssey Imaging System (LI-COR Biosciences, Lincoln, NE, USA). Band intensities from the resulting images were quantified by densitometric analysis using Image Studio Lite software (LI-COR Biosciences, Lincoln, NE, USA). The following primary antibodies were used: mouse monoclonal against adiponectin (Pierce Biotechnology, Rockford, IL, USA); rabbit polyclonal against aP2 (FABP4) (Abcam Inc., Cambridge, MA, USA); rabbit polyclonal against STAT5A (Santa Cruz Biotechnology Inc., Dallas, TX, USA). Goat secondary antibodies against mouse or rabbit IgG, labeled with near infrared dyes, were obtained from LI-COR Biosciences.

### 2.7. Statistics

Statistical analysis was performed using GraphPad Prism software (La Jolla, CA, USA). T-tests were performed with a threshold for statistical significance of *p* < 0.05.

## 3. Results

### 3.1. Groundsel Bush Extract Enhances Neutral Lipid Accumulation and Expression of Adipogenic Marker Genes in Differentiating 3T3-L1 Adipocytes

GB treatment (50 µg/mL) of differentiating 3T3-L1 preadipocytes resulted in induction of several adipogenic genes, as determined by RT-qPCR. Particularly robust (3–4 fold versus control) increases in GLUT4, aP2, adiponectin, and CEBPα mRNA expression were observed (Figure 1A). In a follow-up experiment, differentiating 3T3-L1 adipocytes were treated with 5, 20, or 50 µg/mL of GB, and lipid accumulation was assessed by Oil Red O (ORO) staining. Because it can be technically difficult to detect the improvements in lipid accumulation in cells that are differentiating optimally, we reduced the induction cocktail to half-strength (each component at half concentration). Lipid accumulation under these conditions was enhanced by GB treatment in a dose-dependent manner when compared with vehicle control (Figure 1B,C). An insulin-sensitizing thiazolidinedione, rosiglitazone (Rosi) was used as positive control. The highest dose of GB was not as potent an inducer of lipid accumulation as rosiglitazone. Nonetheless, these results indicate that GB promotes adipogenesis in 3T3-L1 adipocytes.

### 3.2. Groundsel Bush Extract Increases the Protein Expression of Adiponectin and aP2 in Differentiating Adipocytes

**Figure 2:**
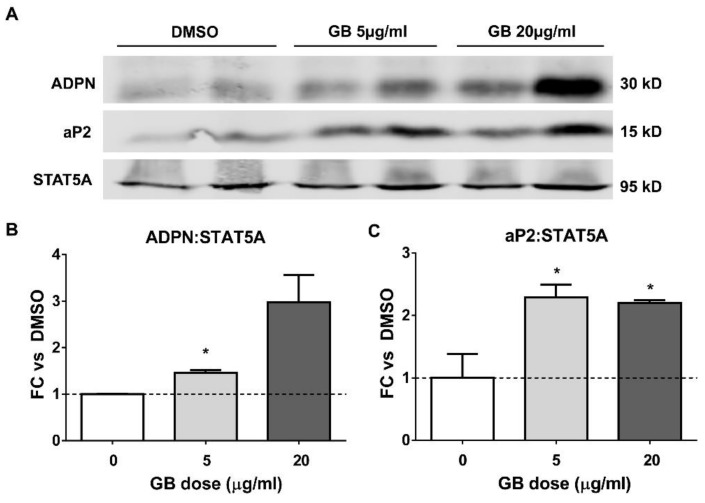
Groundsel bush promotes aP2 and adiponectin (ADPN) expression during the adipogenesis of 3T3-L1 cells. 3T3-L1 preadipocytes were induced to differentiate using standard induction cocktail containing either 5 or 20 µg/mL of groundsel bush (GB). Five days after the induction of differentiation, whole-cell extracts were isolated for Western blot analysis, using 125 μg total protein per lane (**A**); (**B**,**C**) Densitometric quantification of aP2 and ADPN band intensities shown in (**A**). *n* = 2 bands per treatment. * indicates *p* < 0.05 vs. DMSO control. Results shown are from duplicate cell culture wells; this experiment was conducted twice on independent batches of cells.

Western blotting was used to evaluate the expression of adiponectin and aP2 during the differentiation of 3T3-L1 cells in the presence of GB. As shown in Figure 2, the 20 µg/mL dose was most effective in inducing adiponectin expression, while the 5 and 20 µg/mL doses were equally effective on aP2 expression. STAT5A was used as a loading control. Densitometric analysis indicates that GB increases the abundance of both aP2 and adiponectin in differentiating adipocytes.

### 3.3. Groundsel Bush Extract does not Substantially Alter Adiponectin Gene Expression, but Decreases Resistin Gene Expression in a Time-Dependent Manner in Mature 3T3-L1 Adipocytes

**Figure 3:**
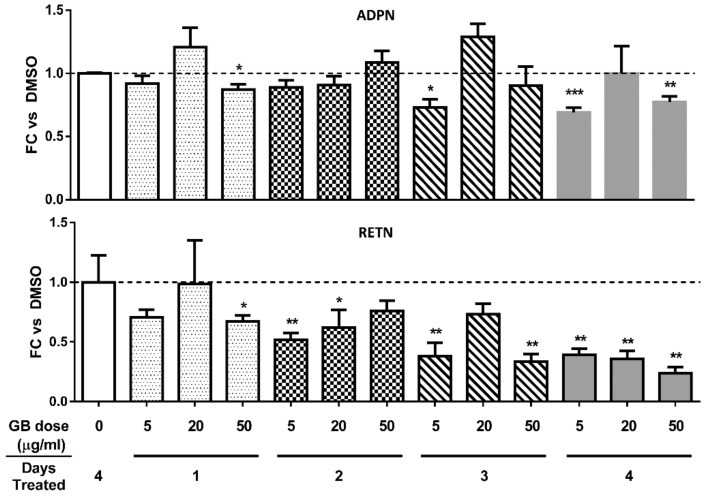
Groundsel bush does not substantially alter adiponectin mRNA, but decreases resistin mRNA levels in mature 3T3-L1 adipocytes. Fully differentiated 3T3-L1 adipocytes were treated with various doses of GB (triplicate cell culture wells for each condition). RNA was isolated, purified, and subjected to RT-qPCR. Target gene quantities were normalized to a reference gene (cyclophilin B). Graphs show fold change (FC) versus the DMSO control as a function of GB dose and number of treatment days for adiponectin (ADPN; top) and resistin (RETN; bottom) gene expression. * indicates *p* < 0.05, ** indicates *p* < 0.01, *** indicates *p* < 0.001 vs. DMSO control.

Adiponectin has been demonstrated to exhibit insulin sensitizing [13,14,15] and anti-inflammatory properties [16,17], and serum levels of adiponectin are negatively correlated with obesity [18]. Hence, we examined the effect of GB on adiponectin gene expression in fully differentiated 3T3-L1 adipocytes. As shown in Figure 3 (top panel), a four-day GB treatment did not induce any time- or dose-dependent changes in adiponectin gene expression. Resistin is an adipokine that is positively associated with adipose tissue inflammation and insulin resistance [19]. GB treatment resulted in diminished gene expression of resistin in a time-dependent fashion (Figure 3, bottom panel), raising the possibility that the extract could have anti-inflammatory and metabolically beneficial effects in adipocytes.

### 3.4. Groundsel Bush Extract Increases Adiponectin Protein Expression in Mature 3T3-L1 Adipocytes in a Time-Dependent Manner

**Figure 4:**
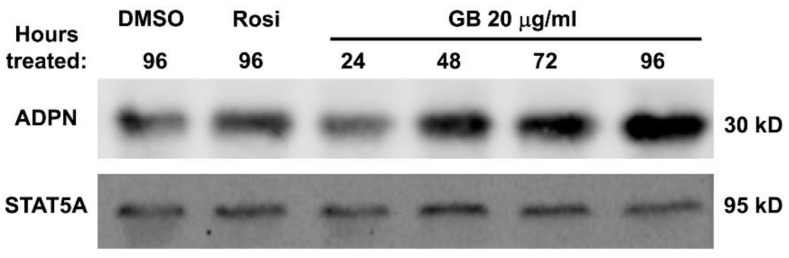
Groundsel bush increases adiponectin expression in mouse adipocytes. Fully differentiated 3T3-L1 adipocytes were treated with vehicle or 1 µM rosiglitazone (Rosi) for 96 h, or with 20 µg/mL groundsel bush extract for the indicated times. Each lane contains 50 μg protein from whole-cell extracts pooled from two biological replicates. Extracts were separated by SDS-PAGE, transferred to nitrocellulose, and subjected to Western blot analysis. Membrane was probed for STAT5A as a loading control. Several independent experiments on different batches of 3T3-L1 adipocytes have shown that GB increases adiponectin expression in a time-dependent manner.

Fully differentiated adipocytes were treated with 20 µg/mL of GB, and adiponectin protein abundance was evaluated by Western blot analysis. Again, rosiglitazone was used for comparison as a positive control. As shown in Figure 4, GB treatment increased adiponectin protein abundance to levels even greater than rosiglitazone treatment in a time-dependent manner over a four-day period of treatment.

### 3.5. Groundsel Bush Extract Attenuates TNF-α-Induced Repression of Adiponectin Expression in 3T3-L1 Adipocytes

**Figure 5:**
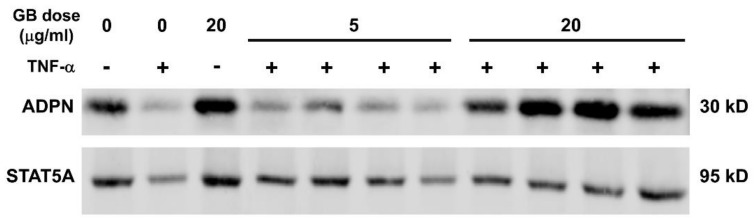
Groundsel bush attenuates TNF-α-induced repression of adiponectin expression in 3T3-L1 adipocytes. Fully differentiated 3T3-L1 adipocytes were treated daily with 0 (DMSO vehicle), 5 or 20 μg/mL GB and 0.5 nM TNF-α (+ condition) or 0.1% BSA vehicle (− condition). After three days, whole-cell extracts were isolated and used for Western blot analysis (50 μg total protein per lane). Membrane was probed for STAT5A as a loading control. Four biological replicates are shown for the GB treatments in the presence of TNF-α.

Inflammation in adipose tissue, mediated by cytokines, such as TNF-α, is known to reduce adiponectin levels and contribute to obesity-induced metabolic dysfunction [20,21,22,23]. Fully differentiated adipocytes were treated with or without 0.5 nM TNF-α and 5 or 20 µg/mL of GB for 3 days, following which whole-cell extracts were collected and used for Western blot analysis. As shown in Figure 5, TNF-α reduced adiponectin protein levels. In addition, GB treatment of 20 µg/mL attenuated the TNF-α-induced repression of adiponectin protein expression. These effects were not observed with the 5 µg/mL dose of GB. 

### 3.6. Groundsel Bush Extract Attenuates LPS-Induced Expression of IL-1β, IL-6, and MCP1, but not TNF-α, in RAW Macrophages

**Figure 6:**
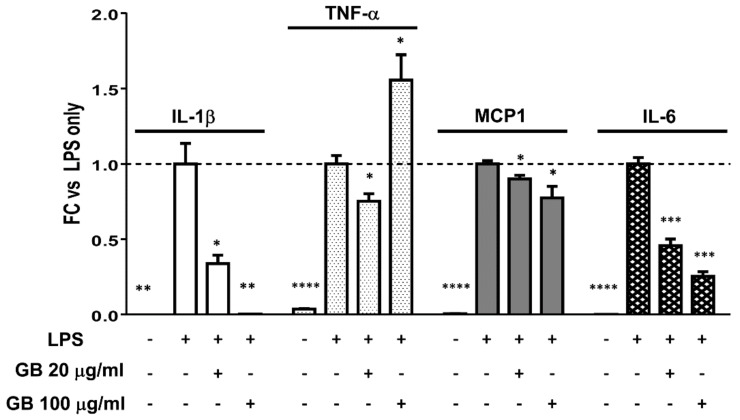
Groundsel bush reduces LPS-induced expression of some, but not all inflammatory genes in RAW macrophages. RAW 264.7 cells were treated for two hours with vehicle (DMSO) or GB at 20 or 100 μg/mL; LPS was then added to a final concentration of 1 μg/mL. Cells were incubated for six additional hours, then harvested for RNA extraction and subsequent RT-qPCR. Target gene quantities were normalized to the reference gene, ubiquitin B. * indicates *p* < 0.05; ** indicates *p* < 0.01; *** indicates *p* < 0.001; **** indicates *p* < 0.0001 vs. LPS-treated controls. *n* = 3 replicate wells per treatment.

Macrophage-derived inflammatory mediators contribute to adipocyte dysfunction in obesity and diabetes. RAW 264.7 macrophages were treated with LPS to induce an inflammatory response, with or without a 2-h GB pre-treatment. Gene expression analyses revealed the expected induction of IL-1β, IL-6, MCP1, and TNF-α mRNA levels with LPS treatment. GB treatment caused a significant dose-dependent reduction in LPS-induced IL-6 and MCP1 expression (Figure 6). As previously reported [12], GB also greatly reduced IL-1β expression (by 66% and >99% at 20 and 100 µg/mL respectively), but not TNF-α expression, which was actually further enhanced at the highest dose of GB.

## 4. Discussion

The indispensable role that is played by adipose tissue in preserving metabolic homeostasis supports the modulation of fat cell function as a valuable therapeutic strategy to ameliorate metabolic dysfunction. Using both in vitro and in vivo experimental approaches, our laboratory has screened and identified botanical extracts that are capable of altering adipocyte development and function [10,11]. The present study examines effects of a botanical extract from *Baccharis halimifolia*, a plant native to Louisiana with a history of use in Creole folk medicine, on fat cell development and function that are likely to be metabolically beneficial. In addition, we have observed anti-inflammatory effects of GB on macrophages that may also contribute to mitigating metabolic dysfunction in adipose tissue, since macrophages in fat tissue are known to mediate insulin resistance and the disruption of endocrine function in adipocytes [24]. To the best of our knowledge, these two lines of evidence (in macrophages and adipocytes) represent the first instance of peer-reviewed research supporting the potential medicinal use of groundsel bush (*Baccharis halimifolia*).

Although obesity is typically a pathological state that results in the expansion of adipose tissue mass through both hypertrophy and hyperplasia, the failure of adipocytes to expand in response to excess energy intake can result in ectopic lipid deposition and insulin resistance [4,25]. There is also evidence that obesity is associated with increased adipocyte turnover due to an elevated rate of cell death [26]. These lines of evidence and many others suggest that adipose tissue expansion associated with enhanced adipogenesis preserves adipose tissue endocrine function and lipid storage capacity, thereby exerting beneficial metabolic effects. Previous work in our laboratory has provided evidence that botanically-derived extracts that promote adipogenesis in vitro could also enhance secretory function in adipocytes and improve glucose homeostasis in mice subjected to diet-induced obesity [10,11]. A preliminary screening experiment (Figure 1A) revealed that GB could induce several adipogenic marker genes, including GLUT4, adiponectin, aP2, CEBPα, fatty acid synthase, and PPARγ, prompting us to further investigate the effects of GB on adipocyte differentiation and function. We observed that GB increases lipid accumulation (Figure 1B,C) as well as the levels of adiponectin and aP2 during adipogenesis of 3T3-L1 cells (Figure 2). Taken together, these data strongly suggest that GB enhances adipocyte development, an effect consistent with improved metabolic function.

In mature adipocytes, our results demonstrate that GB promotes the expression of genes and proteins that are consistent with normal endocrine function and resilience to pro-inflammatory stimuli. Adiponectin, an adipocyte-derived hormone abundant in serum, is known to promote insulin sensitivity in peripheral tissues and to possess anti-inflammatory properties [13,14,15]. Surprisingly, GB did not produce any dose- or time-dependent changes in adiponectin gene expression, and, in fact, reduced its expression slightly in some conditions (Figure 3). It is known, however, that adiponectin is subject to post-transcriptional regulation, and Western blot analysis revealed that GB increased protein levels of adiponectin in fully differentiated adipocytes over a chronic four-day treatment (Figure 4). Resistin, another endocrine hormone secreted by adipose tissue, is associated with inflammation and insulin resistance [27]. GB treatment reduced resistin gene expression in mature adipocytes in a time-dependent manner over a chronic four-day treatment (Figure 3). These in vitro results indicate that GB has the potential to induce metabolically favorable changes in adipocyte endocrine function that may also attenuate inflammation associated with obesity and metabolic syndrome.

To further evaluate the possible anti-inflammatory effects of GB, we subjected mature adipocytes to an inflammatory stimulus and examined adiponectin protein expression in response to this treatment. Adipose tissue is a source of both pro- and anti-inflammatory cytokines, and obesity/type 2 diabetes is often associated with a pro-inflammatory profile. TNF-α, a pro-inflammatory cytokine that is associated with insulin resistance, has been shown to inhibit adiponectin production [28,29,30], and the inhibition of TNF-α can improve glucose homeostasis and increase the serum levels of high-molecular-weight adiponectin [31]. Our results indicate that GB treatment preserved adiponectin protein expression in mature adipocytes exposed to TNF-α. In addition to adipocytes, macrophages are known to reside in adipose tissue depots and to secrete inflammatory cytokines in states of obesity and insulin resistance [32,33]. Treatment of macrophages with GB corroborated previously published findings that GB could greatly reduce LPS-induced expression of IL-1β. In addition, we show here that GB also inhibits the expression of the pro-inflammatory genes MCP1 and IL-6, though not to the same extent as IL-1β. While the highest dose of GB tested actually increased TNF-α gene expression in macrophages, this increase was modest when compared with the dramatic dose-dependent reductions of the other three pro-inflammatory genes assayed. In addition, we show that in mature adipocytes, adiponectin protein expression impaired by TNF-α treatment can be rescued by GB. Taken together, the effects of GB on adipocytes and macrophages presented here provide evidence that this botanical extract could attenuate inflammation and promote an adipokine profile consistent with metabolically beneficial glucose homeostasis and insulin sensitivity. 

Future studies will be needed to assess the potential of developing a GB extract as a supplement capable of promoting metabolic resiliency. These would include assessing the effects of GB on adipocyte functions such as lipolysis and lipogenesis; determining whether GB affects human adipocyte development and endocrine function; and examining whether the in vitro effects described herein translate to positive changes in adipose tissue in vivo.

## 5. Conclusions

The epidemics of obesity and type 2 diabetes urgently require the development of therapeutic strategies that are effective in combating these pathological states. As nutraceuticals, plant-derived nutritional supplements offer potentially attractive complementary components of a multifactorial approach to alleviating the burden of metabolic syndrome and its associated pathologies. Given the major role of healthy adipose tissue in regulating insulin sensitivity and glucose homeostasis, fat cells are a viable target for therapies that are aimed at enhancing metabolic resilience in an obesogenic environment. The in vitro studies reported herein provide evidence that an extract derived from groundsel bush, a botanical previously undescribed in the biomedical literature, stimulates adipocyte development, enhances gene and protein expression of adiponectin, and shows potential in promoting an anti-inflammatory adipokine profile. Taken together, the results of these novel experiments support the hypothesis that GB has potential to exert beneficial effects on insulin sensitivity, inflammation, and glucose homeostasis via the modulation of adipocyte function. Future research aimed at discovering potential bioactive compounds in this botanical and better understanding the biochemical mechanisms by which it exerts its effects could provide a platform from which to extend studies to in vivo models of obesity and insulin resistance. 

## Figures and Tables

**Table 1:** Primer sequences for genes analyzed by qPCR.

Gene Name and Symbol	Forward Primer, 5′-3′	Reverse Primer, 5′-3′
adiponectin (adpn)	AAAAGGGCTCAGGATGCTACTG	TGGGCAGGATTAAGAGGAACA
resistin (retn)	CCTTTTCTTCCTTGTCCCTGA	TGTCCAGCAATTTAAGCCAATG
interleukin-1 (il1b)	GACCTGTTCTTTGAAGTTGACG	CTCTTGTTGATGTGCTGCTG
tumor necrosis factor-alpha (tnf)	AGACCCTCACACTCAGATCA	TCTTTGAGATCCATGCCGTTG
monocyte chemoattractant protein 1 (ccl2)	GCAGAGAGCCAGACGGGAGGA	TGGGGCGTTAACTGCATCTGG
interleukin-6 (il6)	TCCTCTCTGCAAGAGACTTCCATCC	AAGCCTCCGACTTGTGAAGTGGT
cyclophilin B (ppib)	AGCAAGTTCCATCGTGTCATC	CCGTAGTGCTTCAGCTTGA
ubiquitin B (ubb)	CCAGTGGGCAGTGATGG	GCTTACCATGCAACAAAACCT
